# Redefining lightweight vision models for healthcare AI

**DOI:** 10.3389/frai.2026.1824634

**Published:** 2026-05-29

**Authors:** Linus Lee, Zhibin Feng, Jen Hong Tan, Chiaw-Ling Chng

**Affiliations:** 1 Data Science and Artificial Intelligence Lab, Singapore General Hospital, Singapore, Singapore; 2 Department of Endocrinology, Singapore General Hospital, Singapore, Singapore

**Keywords:** Grouped Query Attention (GQA), ImageNet dataset, lightweight model, masked AutoEncoder, MedMNIST dataset, Mixture-of-Experts (MoE), SwiGLU feedforward network, Vision Transformer (ViT)

## Abstract

**Background:**

Vision models for medical imaging often require tens of millions of parameters, raising questions about whether architectural efficiency can be achieved without sacrificing classification performance. We introduce MedLiT-seed (2.1 Million parameters) and MedLiT-nano (0.75 Million parameters), two ultra-lightweight vision transformers designed for efficient and scalable medical image analysis.

**Methods:**

MedLiT employs a streamlined Mixture-of-Experts (MoE) architecture with SwiGLU feedforward networks, grouped query attention, and depth-wise scaling. Models were pre-trained using masked autoencoding on ImageNet and MedMNIST, followed by fine-tuning on 12 MedMNIST 2D subsets. We evaluated performance across multiple configurations and compared against benchmark models including ResNet, MedViT, and AutoML systems.

**Results:**

MedLiT-seed achieved the highest Area Under Curve (AUC) on 4 subsets and second-highest on 2 others, outperforming models with 10–20× more parameters. MedLiT-nano achieved results comparable to, and even exceeding, ResNet-18 and AutoML baselines in several subsets. Transfer learning from ImageNet significantly improved convergence and generalization. Increasing embedding size yielded greater performance gains than increasing expert count.

**Conclusion:**

MedLiT demonstrates that MoE-based token routing represents a viable architectural pathway for achieving competitive accuracy relative to its floating-point operations (FLOP) across diverse medical imaging modalities on the order of 2M parameters. These results suggest that selectively routing computation through specialised experts, rather than scaling model size, can serve as an effective design principle for more compact medical vision models. Such architecture can be utilised for low-resource clinical environments and scalable fine-tuning across diverse healthcare tasks, though limitations on multi-label tasks highlight clear directions for future architectural refinement.

## 1 Introduction

Recent advances in deep learning have enabled the development of foundation models capable of generalizing across diverse medical imaging tasks (van Veldhuizen et al., 2025). However, the computational demands of these models—often exceeding tens of millions of parameters—pose significant barriers to their deployment in real-world clinical settings, particularly in resource-constrained environments such as rural clinics, mobile health platforms, and embedded systems. Their size raises a practical research question: how much of that performance is attributable to scale, and how much to architectural design choices?

In this study, we present MedLiT, a family of ultra-lightweight vision transformers designed specifically for medical imaging (Tan, 2024) via a compute-efficient architectural lens. MedLiT-seed (2.1M parameters) and MedLiT-nano (0.75M parameters) are built around the hypothesis that selective computational routing, rather than uniform scaling, can serve as an effective design principle for compact medical vision models. By integrating Mixture-of-Experts (MoE) layers (Shazeer et al., 2017) with SwiGLU feedforward networks (Shazeer, 2020), grouped query attention (Ainslie et al., 2023) and progressive depth-wise scaling, MedLiT streamlines rather than augments the transformer backbone with its novel architectural strategy.

To test this, we conduct a comprehensive series of experiments on the MedMNIST v2 2D benchmark (Yang et al., 2023), comparing MedLiT against conventional Convolutional Neural Networks (CNN) (O’Shea and Nash, 2015), hybrid CNN and vision transformer (ViT) architectures (Long, 2024; Wu et al., 2021), State Space Models (SSM) (Gu et al., 2021) and Automated Machine Learning (AutoML) systems (Shen et al., 2018; Zöller and Huber, 2021). We pay particular attention to accuracy-per-FLOP trade-offs across model families and further analyse architectural design decisions including embedding size scaling, expert count, and pre-training strategy.

## 2 Related works

Medical image analysis has undergone a series of architectural paradigm shifts over the past decade, especially in the field of medical imaging. This section traces that evolution and situates MedLiT within it.

### 2.1 CNN-based architectures

The Introduction of the U-shaped Network architecture (U-Net) establishes the encoder-decoder paradigm as the dominant framework for medical image segmentation, leveraging skip connections to preserve spatial detail across scales (Zhang C. et al., 2024). For classification tasks, ResNet demonstrated that residual connections enable stable training of deep networks by facilitating gradient flow in deep networks (He et al., 2016), resulting in its wide adoption in clinical AI research for its simplicity, robustness and ease of deployment (Xu et al., 2023). ResNet-18 and ResNet-50 in particular have proven to be reliable baselines for MedMNIST, owing to their consistent performance across diverse, low-resolution multi-modal datasets. However, CNN-based models are fundamentally constrained by their local receptive fields, limiting their ability to model long-range spatial dependencies that are often diagnostically relevant in medical images.

### 2.2 Transformer-based and hybrid architectures

The success of ViT in capturing global dependencies via self-attention prompted rapid adaptation to the medical domain, such as its application to breast cancer screening (Gheflati and Rivaz, 2022). Hybrid Transformer-CNN architectures extended this further by combining the complementary strengths of both paradigms. TransUNet integrates both Transformer modules and 3D CNNs to effectively capture local and global characteristics, and outperformed previous models for 3D brain tumor segmentation (Chen et al., 2021). Similarly, MedViTV1 combines convolutional blocks for local feature extraction with transformer modules for global context, proving particularly effective on medical images which often contain fine-grained structural details alongside broader contexture patterns (Manzari et al., 2023). Building on this, MedViTV2 (Manzari et al., 2026) incorporates Kolmogorov-Arnold Network (KAN) layers to enhance representation learning and improve performance on structurally complex datasets (Liu et al., 2024). Despite their representational power, transformer-based models are limited by the quadratic complexity of the self-attention mechanism with respect to input sequence length, resulting in high parameter and FLOP counts that can pose challenges for resource-constrained research settings and rapid clinical iterations.

### 2.3 Efficiency-driven architectures

More recently, the field has shifted attention towards architectures that maintain competitive performance under tighter computational budgets (Zhang H. et al., 2024). SSM’s such as Mamba-based architectures emerged as a compelling alternative to self-attention by capturing long-range dependencies with linear rather than quadratic complexity (Gu and Dao, 2024). MedMamba demonstrated that SSM-based models can match or exceed transformer-level performance on MedMNIST with a significantly reduced model footprint, making them well-aligned with resource-constrained deployment scenarios (Yue and Li, 2024). MedMamba-T/S/B variants are included in our comparisons to evaluate MedLiT across this efficiency-accuracy trade-off. Concurrently, MoE routing has gained traction as a strategy for scaling model capacity without proportional increases in per-sample computation by conditionally activating a subset of parameters for each input (Shazeer et al., 2017). While MoE has recently seen adoption in object detection architectures such as YOLO-Master (Lin et al., 2025), or MoE-Mamba which combines MoE with Mamba architecture (Pióro et al., 2024), its application to lightweight vision architectures for medical image classification remains underexplored.

### 2.4 AutoML systems

AutoML systems, such as AutoKeras (Jin et al., 2019) and Google AutoML Vision (Bisong, 2019), offer automated pipelines for neural architecture search and hyperparameter optimization. These tools enable non-expert users to achieve reasonable classification results without manual model design (Shen et al., 2018; Zöller and Huber, 2021). However, their lack of architectural transparency and reproducibility poses challenges for rigorous evaluation and clinical validation. Despite these limitations, AutoML models serve as useful baselines for assessing the performance of lightweight and interpretable models like MedLiT. As per the MedMNISTv2 study, AutoKeras is designated as the representative of open-source AutoML tools for deep learning while Google AutoML is designated as the representative of commercial black-box AutoML tools with deep learning empowered (Yang et al., 2023).

### 2.5 Positioning MedLiT

This progression from handcrafted CNNs to attention-based hybrids, efficiency-driven SSMs and automated systems reveals a persistent tension between representational capacity and computational cost. Lightweight transformer designs and MoE-based conditional computation represent a complementary response to this tension, yet prior work has not unified them in an architecture targeting medical image classification. MedLiT addresses this gap directly: by integrating MoE routing into a lightweight vision transformer backbone and fine-tuning on the MedMNISTv2 benchmark, it achieves performance comparable to models of substantially larger parameter and FLOP counts, demonstrating that competitive representational capacity need not come at the cost of computational efficiency.

## 3 Methods

### 3.1 Model architecture

MedLiT is a lightweight vision transformer built on a Mixture-of-Experts (MoE) framework following the implementation specified in (Tan, 2024). Its encoder comprises 9 transformer layers organized into three stages, with each stage containing a progressively increasing number of experts (3, 4, and 5 respectively) per layer. We follow the structural design laid out in (Tan, 2024). Each expert is implemented as a SwiGLU feedforward network (Shazeer, 2020) where V and W2 are shared across a layer, and a learned gating function selects the top-2 experts per token, enabling sparse activation for more efficient computation (Shazeer et al., 2017). To further reduce parameter count, grouped query attention (Tan, 2024; Ainslie et al., 2023) is used with 6 attention heads split into 3 groups. The hidden dimension of each feedforward layer is progressively scaled down from 81 to 27 across the depth of the network (Tan, 2024).

**Figure 1:**
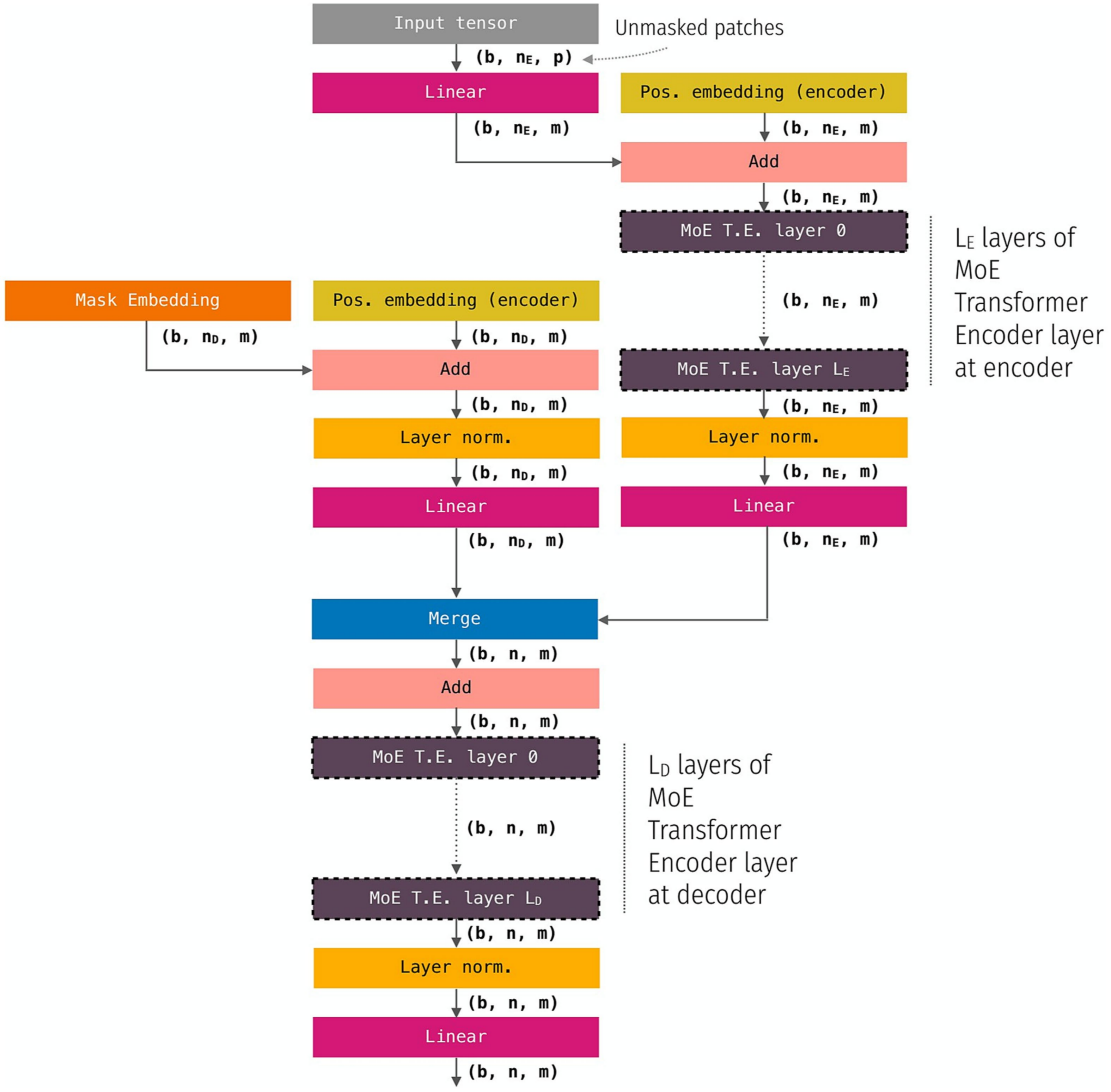
Architecture overview of MedLiT.

During pre-training, a lightweight decoder consisting of 4 transformer layers with 4 experts each is appended to the encoder. This decoder is used solely for reconstructing masked image patches and is discarded during fine-tuning. An overview of MedLiT’s general architecture is provided in Figure 1 for reference. Additionally, a more detailed diagram detailing the different layers of MedLiT-seed is attached in the Figure A1.

### 3.2 Datasets

For this study, we utilized two large datasets: *MedMNISTv2* (Yang et al., 2023) and *ImageNet* (ILSVRC 2012–2017) (Deng et al., 2009). MedMNIST is a large-scale, MNIST-like collection of standardized biomedical images. It comprises 12 2D and 6 3D subsets spanning a variety of imaging modalities, including X-ray, OCT, ultrasound, and CT. Our experiments focused on the 12 2D subsets, each resized to 224 × 224 pixels, totalling approximately 708,000 images. These subsets encompass a diverse range of classification tasks, including binary, multi-class, multi-label, and ordinal regression (Yang et al., 2023).

To support transfer learning and evaluate generalization, we also incorporated the ImageNet Large Scale Visual Recognition Challenge 2012–2017 dataset, which contains 1.4 million annotated natural images across 1,000 object categories (Russakovsky et al., 2015). This dataset was used for both pre-training and fine-tuning in selected experiments to help MedLiT learn general-purpose visual representations prior to domain-specific adaptation.

For both datasets, we use their default training/validation/testing data splits. The use of these data splits are further described in the following sections on pre-training and fine-tuning.

### 3.3 Pre-training strategy

Pre-training is conducted using a masked autoencoding framework (He et al., 2022). Input images are passed through a transformation pipeline where they are first resized to 256 × 256 and randomly horizontally flipped (*p* = 0.5). As some MedMNIST subsets are a 1 channel grayscale, we replicate the single channel from 1 to 3. We then normalize all images with a mean of 0.5 and standard deviation (s.d.) of 0.5 across all 3 channels for MedMNIST images, or the ImageNet mean and s.d. for ImageNet images [mean = (0.485, 0.456, 0.406), s.d. = (0.229, 0.224, 0.225)].

**Figure 2:**
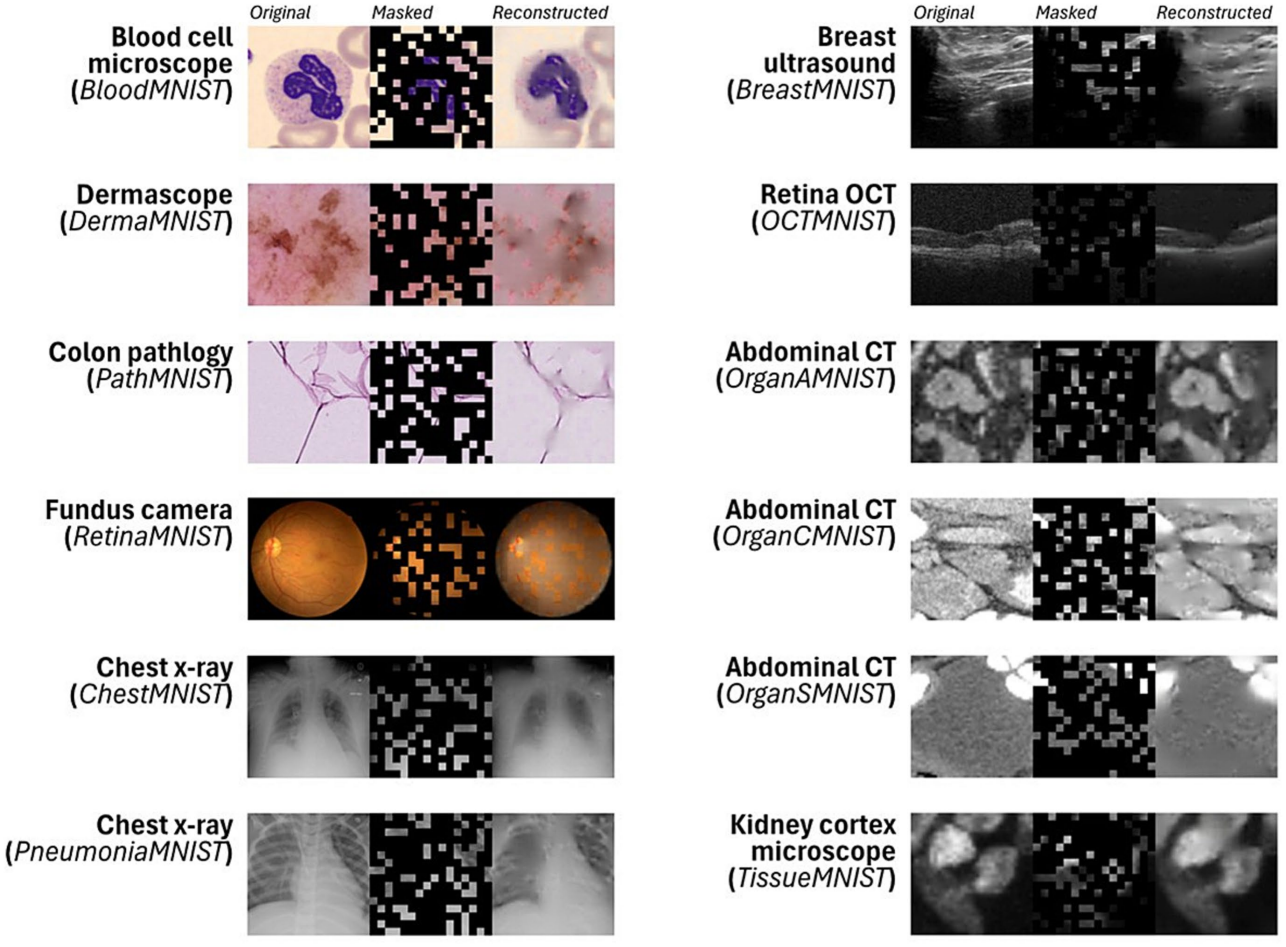
Original, masked, and reconstructed images for each MedMNIST subset and their respective imaging modalities, experiment 10.

Input images are then divided into non-overlapping 16 × 16 patches, yielding 256 tokens per image. A class token is prepended, and 75% of the patch tokens are randomly masked. The encoder is trained to reconstruct the original image from the remaining visible patches. Each patch is embedded into an n-dimensional vector, with n referring to the image embedding size. MedLiT-nano and MedLiT-seed have an embedding dimension of 108 and 216, respectively. An example of masked autoencoding for MedMNIST during pre-training in Experiment 10 is depicted below in Figure 2 (see Table A2 for experiment conditions).

Three loss components are used during pre-training:

- Reconstruction loss: Mean squared error between original and reconstructed patch embeddings (He et al., 2022).
- MoE balancing loss: Encourages uniform utilization of experts across tokens (Shazeer et al., 2017).
- Channel consistency loss: Promotes similarity across RGB channels, particularly useful for grayscale medical images converted to RGB. This term is weighted by *λ* = 0.1.

Channel consistency loss is particularly effective for datasets originating from single-channel grayscale images that were converted to a three-channel RGB format via channel replication, and the weight mitigates undesired bias introduced for naturally coloured images.

Pre-training is performed on both MedMNIST/ImageNet, using the AdamW optimizer with an initial learning rate of 1 × 10^−4^, a cosine decay schedule (Figure A2) and mixed precision training. Gradient scaling is applied dynamically to maintain numerical stability.

Pre-training on MedMNIST in the case of MedLiT-nano involves concatenating the train splits of the 12 MedMNIST 2D subsets into a single dataset. Pre-training on ImageNet in the case of MedLiT-seed utilizes only the train split of the dataset. For both cases, the encoder is saved only after 120 epochs of pre-training.

### 3.4 Fine-tuning strategy

Fine-tuning involves replacing the decoder with a lightweight linear classification head that maps the encoder’s output to the task-specific label space. No activation function is applied in the head, as it is handled by the loss function.

Fine-tuning on ImageNet for MedLiT-seed involves the training and validation split of the ImageNet dataset. The pre-trained encoder is further trained on the training split before testing with the validation subset, and we save the model with the highest validation accuracy for the final fine-tuning step with MedMNIST.

Fine-tuning on MedMNIST for MedLiT-nano and -seed involves the training and validation splits of the 12 MedMNIST 2D subsets. For each subset, the pre-trained encoder is further trained on the training split of the MedMNIST subset before being tested on the validation split after every epoch. The model with the highest validation accuracy is saved for each subset. This model is finally used on the testing split of that subset to obtain the final test accuracy.

For experiments (see Table A2) where fine-tuning on ImageNet is involved, input images are resized to 256 × 256 and horizontally flipped (*p* = 0.5) before being normalized with the ImageNet mean and standard deviation [mean = (0.485, 0.456, 0.406), s.d. = (0.229, 0.224, 0.225)].

For standard fine-tuning on MedMNIST, input images are resized to 256 × 256 before normalisation with a mean = 0.5 and s.d. = 0.5 across all 3 channels.

Optimization is again performed using AdamW, with learning rates tailored to each MedMNIST subset based on empirical stability of 1/3/5 × 10^−4^ (Table A1), and a base learning rate of 5 × 10^−4^ for ImageNet. These learning rates are additionally scaled by batch size and gradient accumulation steps. A layer-wise learning rate decay (factor 0.9) is applied to preserve pre-trained features in earlier layers. Fine-tuning also uses mixed precision training, gradient clipping and a cosine decay schedule (Figure A3).


$$L_{\text{focal}}(L_{\text{base}})=1[p_{t}<\tau ]\cdot {\left(1-p_{t}\right)}^{\gamma }\cdot L_{\text{base}}$$ (1)

$${ℒ}_{\text{multi}-\text{class}}={ℒ}_{\text{focal}}({ℒ}_{\mathrm{CE}})$$ (2)

$${ℒ}_{\text{multi}-\text{label}}=0.5\cdot {ℒ}_{\mathrm{BCE}}+0.5\cdot {ℒ}_{\text{focal}}({ℒ}_{\mathrm{BCE}})$$ (3)

Equation 1: Fine-tuning loss.

Loss functions vary by task type. For multi-class tasks where each image is assigned a single label, we apply a modified Focal Loss over the cross-entropy logits (Equation 1, lines 1, 2). A hard mask zeroes out the loss contribution of any sample with confidence pt ≥ 0.8, ensuring training focuses exclusively on uncertain samples.

For multi-label tasks (ChestMNIST) where each image may belong to multiple class labels simultaneously, we treat each label as an independent binary problem. We use a composite loss that equally weighs standard Binary Cross-Entropy (BCE) with Focal Loss (Equation 1, line 3). The BCE term provides a stable gradient signal across all labels and is supplied a per-label positive weight vector *α*, derived from the inverse frequency of positive samples in the training set, to correct for class imbalance. The Focal term further down-weights easy negatives in the input to improve sensitivity to rare pathologies.

In both cases, we add a MoE balancing loss to encourage uniform expert utilisation during fine-tuning (Shazeer et al., 2017).

### 3.5 Experimental design

To evaluate the robustness and scalability of MedLiT, we conducted 14 experiments (E1–E14) exploring various architectural and training configurations:

- *E1–E4*: Baseline experiments with MedLiT-nano (0.75M parameters), varying pre-training duration (40 vs. 120 epochs) and fine-tuning batch size (32, 64, 128).
- *E5–E6*: Transfer learning experiments incorporating ImageNet pre-training, with E6 adding an intermediate MedMNIST pre-training stage.
- *E7–E8*: Scaling experiments that doubled the number of experts in the encoder (E7) and both encoder and decoder (E8).
- *E9–E11*: Embedding dimension experiments, increasing from 108 to 128 (E9) and 216 (E10), with E11 combining increased embedding size and expert count.
- *E12–E14*: Consolidated experiments using MedLiT-seed (2.1M parameters), combining ImageNet pre-training and fine-tuning with MedMNIST fine-tuning. E14 further increased expert count to 2.5M parameters.

These experiments helped to assess the impact of architectural scaling, transfer learning, and training schedules on model performance. Results from each configuration were evaluated using test accuracy (ACC) and AUC across all 12 MedMNIST subsets, providing a comprehensive view of MedLiT’s capabilities and limitations.

All experiments are conducted on a NVIDIA H100 NVL GPU with 80GB of GPU RAM. A more in-depth description of the model configurations and training schedules used across all experiments is listed in the Appendix under Table A2.

To promote transparency and facilitate reproducibility, we provide the evaluation code used to assess MedLiT-seed on all 2D subsets of the MedMNIST benchmark, along with trained model weights, through a public GitHub repository.^1^ These resources enable reproduction of the reported accuracy and AUC metrics under the evaluation protocols described in this study.

## 4 Results

As MedLiT is designed for lightweight deployment, our training configuration differs from the standardised MedMNIST benchmark protocol (Yang et al., 2023). Consequently, reported results from baseline models are taken directly from their respective publications and may not reflect performance under identical training conditions. Rather than a direct benchmark comparison, we present our results in the context of parameter efficiency, examining the trade-off between model size and performance.

Among the 14 experiments, Experiment 2 (E2) and Experiment 12 (E12) are used to represent MedLiT-nano and MedLiT-seed, respectively, based on their superior performance relative to other configurations under comparable conditions.

E2 corresponds to MedLiT-nano trained using a standard pre-training–fine-tuning schedule on MedMNIST with an image embedding dimension of 108. This configuration consistently outperformed other MedLiT-nano variants employing alternative training schedules or modified hyperparameters, including different epoch counts and fine-tuning batch sizes.

E12 corresponds to MedLiT-seed trained via pre-training on ImageNet, followed by fine-tuning on ImageNet and MedMNIST, with an image embedding dimension of 216. This experiment achieved the strongest performance across all MedMNIST subsets, outperforming comparable configurations that varied pre-training batch size or increased the number of experts.

**Figure 3:**
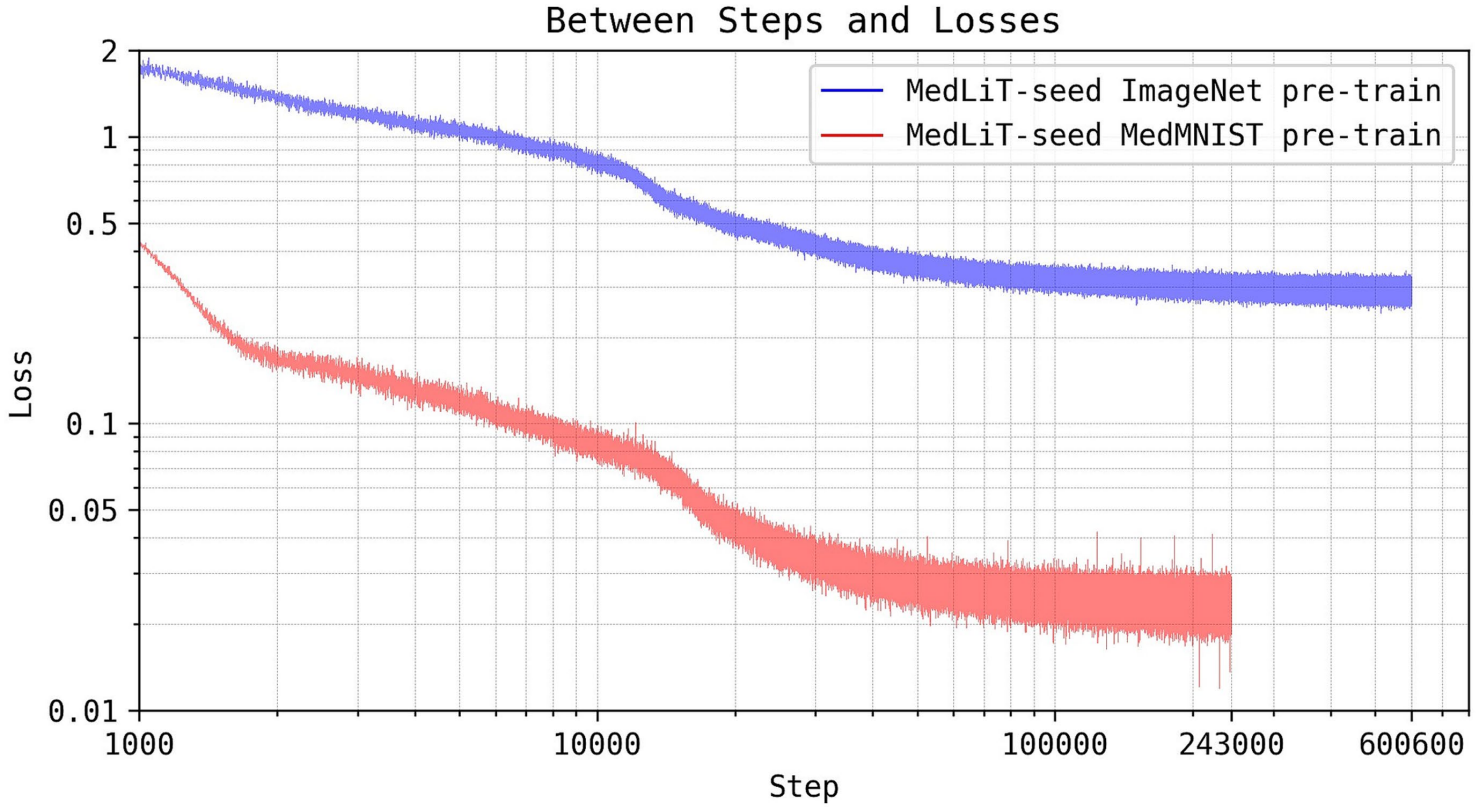
Pre-training loss for MedLiT-seed on MedMNIST (E10) and ImageNet (E12).

Given that MedLiT-seed represents the strongest-performing model, we highlight its pre-training loss on MedMNIST from Experiment 10 and on ImageNet from Experiment 12 in Figure 3. A complete summary of results from all 14 experiments is provided in the Table A2.

We present the test set AUC and ACC for the 12 subsets for both MedLiT-nano and MedLiT-seed in Table 1. Both AUC and ACC are accompanied by 95% confidence intervals in the brackets, derived via non-parametric bootstrap resampling (1,000 iterations) of test set predictions. Each bootstrap iteration resampled the test set with replacement and recomputed AUC/ACC, and the 2.5th and 97.5th percentiles of the resulting distribution are taken as the confidence intervals bounds. ChestMNIST AUC is macro-averaged across its 14 pathology labels. Full multi-seed retraining was not feasible given the breadth of experimental configurations evaluated, 14 ablation settings across 12 subsets, within the available revision period. Accordingly, variance arising from stochastic training is not captured by the reported intervals and should be considered a limitation of the current evaluation. We acknowledge this as a limitation and recommend multi-seed training and evaluation in future comparisons.

**Table 1:** Test set AUC and ACC for MedMNIST.

Subset	MedLiT-nano		MedLiT-seed	
	AUC	ACC	AUC	ACC
PathMNIST	0.9836 (0.9819–0.9853)	0.8617 (0.8538–0.8699)	0.9976 (0.9972–0.9981)	0.9532 (0.9483–0.9578)
ChestMNIST	0.7290 (0.7224–0.7351)	0.8163 (0.8140–0.8188)	0.7819 (0.7754–0.7878)	0.8286 (0.8264–0.8308)
DermaMNIST	0.9246 (0.9109–0.9366)	0.7736 (0.7551–0.7905)	0.9653 (0.9603–0.9702)	0.8299 (0.8135–0.8454)
OctMNIST	0.9619 (0.9545–0.9687)	0.8010 (0.7750–0.8260)	0.9744 (0.9676–0.9809)	0.8590 (0.8370–0.8800)
PneumoniaMNIST	0.9550 (0.9400–0.9696)	0.8526 (0.8253–0.8798)	0.9850 (0.9760–0.9922)	0.8910 (0.8670–0.9151)
RetinaMNIST	0.7444 (0.7052–0.7819)	0.5500 (0.5000–0.5951)	0.8006 (0.7659–0.8338)	0.5975 (0.5500–0.6475)
BreastMNIST	0.8388 (0.7539–0.9175)	0.8397 (0.7821–0.8912)	0.9175 (0.8672–0.9641)	0.8974 (0.8462–0.9423)
BloodMNIST	0.9982 (0.9974–0.9989)	0.9725 (0.9673–0.9775)	0.9993 (0.9985–0.9998)	0.9860 (0.9822–0.9898)
TissueMNIST	0.9295 (0.9235–0.9269)	0.6768 (0.6727–0.6809)	0.9379 (0.9363–0.9395)	0.7207 (0.7169–0.7249)
OrganAMNIST	0.9932 (0.9923–0.9942)	0.9369 (0.9335–0.9405)	0.9991 (0.9989–0.9992)	0.9646 (0.9619–0.9671)
OrganCMNIST	0.9901 (0.9887–0.9914)	0.9030 (0.8965–0.9094)	0.9976 (0.9972–0.9980)	0.9458 (0.9411–0.9507)
OrganSMNIST	0.9685 (0.9664–0.9707)	0.7732 (0.7644–0.7821)	0.9822 (0.9811–0.9833)	0.8161 (0.8079–0.8243)
Mean Across Subset	0.9181	0.8131	0.9449	0.8575

### 4.1 Parameter efficiency comparison

**Figure 4:**
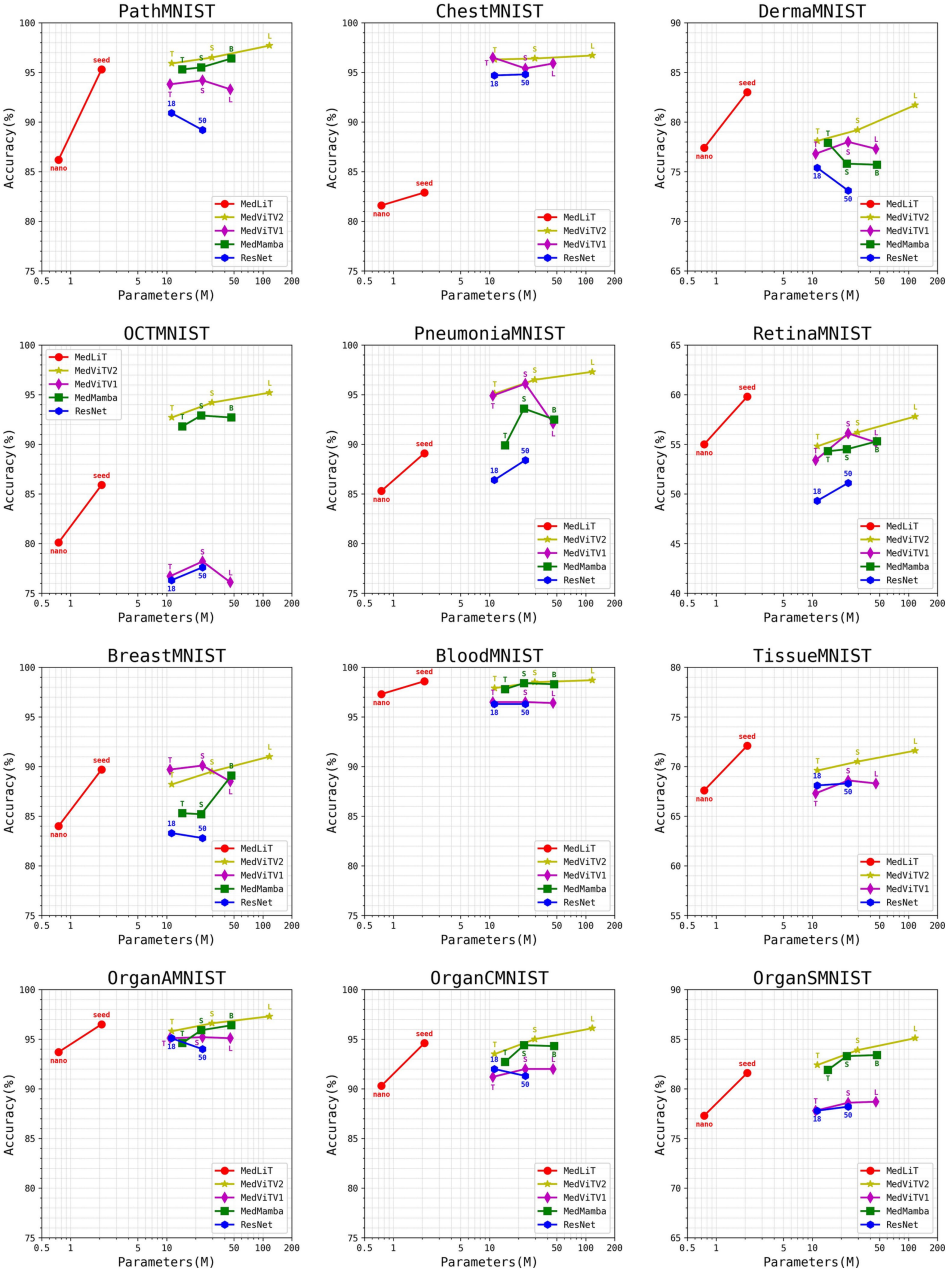
Performance-to-size comparison between MedLiT and benchmark models on MedMNIST.

To evaluate the effectiveness of MedLiT-seed and MedLiT-nano, we conducted a performance-to-size comparison against a diverse set of benchmark models on the MedMNISTv2 2D subsets. These benchmarks include residual networks (ResNet-18 and ResNet-50), hybrid CNN-transformer architectures (MedViTV1 and MedViTV2 T/S/L variants), AutoML systems (AutoKeras and Google AutoML), and SSM models (MedMamba-T/S/B). We first compare model accuracy relative to parameter count on a logarithmic scale, excluding AutoKeras and Google AutoML, for 12 subsets in Figure 4.

A comprehensive overview of MedLiT-nano and MedLiT-seed compared to our model benchmarks for the MedMNIST 2D datasets are shown below in Table 2, using AUC and ACC. Apart from ResNet-18 and ResNet-50 where the total FLOP count is derived from their own study (He et al., 2016), the following statistics (Model parameters, FLOPs, AUC, ACC) are obtained from the MedViTV2 study (Manzari et al., 2026), representing the most recent model used as a benchmark. The FLOP count for MedViTV2-L is an estimate from the study as a concrete value was not provided. A more direct comparison between both sizes of MedLiT and the best performing model amongst the benchmarks, MedViTV2-L, is highlighted in Table 3. Cases where MedLiT-seed outperforms MedViTV2-L are in bold.

**Table 2:** Benchmark comparison with AUC and ACC.

Methods	Params (M)	FLOPs (G)	Path (107,180)		Chest (112,120)		Derma (10,015)		OCT (109,309)		Pneumonia (5,856)		Retina (1,600)	
			AUC	ACC	AUC	ACC	AUC	ACC	AUC	ACC	AUC	ACC	AUC	ACC
ResNet-18 (224) (He et al., 2016)	11.3	1.8	0.989	0.909	0.773	0.947	0.920	0.754	0.958	0.763	0.956	0.864	0.710	0.493
ResNet-50 (224) (He et al., 2016)	23.5	3.8	0.989	0.892	0.773	0.948	0.912	0.731	0.958	0.776	0.962	0.884	0.716	0.511
AutoKeras (Jin et al., 2019)	–	–	0.959	0.834	0.742	0.937	0.915	0.749	0.955	0.763	0.947	0.878	0.719	0.503
Google AutoML (Bisong, 2019)	–	–	0.944	0.728	0.778	0.948	0.914	0.768	0.963	0.771	0.991	0.946	0.750	0.531
MedMamba-T (Yue and Li, 2024)	14.5	4.5	0.997	0.953	–	–	0.917	0.779	0.992	0.918	0.965	0.899	0.747	0.543
MedMamba-S (Yue and Li, 2024)	22.8	6.1	0.997	0.955	–	–	0.924	0.758	0.991	0.929	0.976	0.936	0.718	0.545
MedMamba-B (Yue and Li, 2024)	47.1	13.4	0.999	0.964	–	–	0.925	0.757	0.996	0.927	0.973	0.925	0.715	0.553
MedViTV1-T (Manzari et al., 2023)	31.1	11.7	0.994	0.938	0.786	0.965	0.914	0.768	0.961	0.767	0.993	0.949	0.752	0.534
MedViTV1-S (Manzari et al., 2023)	44.4	16.7	0.993	0.942	0.791	0.954	0.937	0.780	0.960	0.782	0.995	0.961	0.773	0.561
MedViTV1-L (Manzari et al., 2023)	57.6	21.6	0.984	0.933	0.805	0.959	0.920	0.773	0.945	0.761	0.991	0.921	0.754	0.552
MedViTV2-T (Manzari et al., 2026)	12.3	5.1	0.998	0.959	0.791	0.963	0.931	0.781	0.993	0.927	0.995	0.951	0.761	0.548
MedViTV2-S (Manzari et al., 2026)	29.6	7.6	0.998	0.965	0.803	0.964	0.946	0.792	0.994	0.942	0.996	0.965	0.780	0.562
MedViTV2-L (Manzari et al., 2026)	117.3	~26	0.999	0.977	0.823	0.967	0.950	0.817	0.996	0.952	0.997	0.973	0.785	0.578
MedLiT-nano	0.7	0.6	0.984	0.862	0.729	0.816	0.925	0.774	0.962	0.801	0.955	0.853	0.744	0.550
MedLiT-seed	2.1	1.6	0.998	0.953	0.782	0.829	0.965	0.830	0.974	0.859	0.985	0.891	0.801	0.598

Methods	Params (M)	FLOPs (G)	Breast (780)		Blood (17,092)		Tissue (236.386)		OrganA (58,830)		OrganC (23,583)		OrganS (25,211)	
			AUC	ACC	AUC	ACC	AUC	ACC	AUC	ACC	AUC	ACC	AUC	ACC
ResNet-18 (224) (He et al., 2016)	11.3	1.8	0.891	0.833	0.998	0.963	0.933	0.681	0.998	0.951	0.994	0.920	0.974	0.778
ResNet-50 (224) (He et al., 2016)	23.5	3.8	0.889	0.828	0.998	0.963	0.933	0.683	0.997	0.940	0.993	0.913	0.975	0.782
AutoKeras (Jin et al., 2019)	–	–	0.841	0.790	0.998	0.962	0.934	0.677	0.997	0.937	0.993	0.914	0.974	0.772
Google AutoML (Bisong, 2019)	–	–	0.906	0.859	0.998	0.965	0.933	0.675	0.997	0.937	0.992	0.904	0.970	0.769
MedMamba-T (Yue and Li, 2024)	14.5	4.5	0.825	0.853	0.999	0.978	–	–	0.998	0.946	0.997	0.927	0.982	0.819
MedMamba-S (Yue and Li, 2024)	22.8	6.1	0.806	0.853	0.999	0.984	–	–	0.999	0.959	0.997	0.944	0.984	0.833
MedMamba-B (Yue and Li, 2024)	47.1	13.4	0.849	0.891	0.999	0.983	–	–	0.999	0.964	0.997	0.943	0.984	0.834
MedViTV1-T (Manzari et al., 2023)	31.1	11.7	0.923	0.897	0.998	0.965	0.931	0.673	0.998	0.951	0.993	0.912	0.973	0.778
MedViTV1-S (Manzari et al., 2023)	44.4	16.7	0.925	0.901	0.998	0.965	0.938	0.686	0.998	0.952	0.994	0.920	0.975	0.786
MedViTV1-L (Manzari et al., 2023)	57.6	21.6	0.918	0.885	0.998	0.964	0.937	0.683	0.998	0.951	0.994	0.920	0.975	0.787
MedViTV2-T (Manzari et al., 2026)	12.3	5.1	0.944	0.882	0.999	0.979	0.936	0.696	0.998	0.958	0.997	0.935	0.985	0.824
MedViTV2-S (Manzari et al., 2026)	29.6	7.6	0.947	0.895	0.999	0.985	0.939	0.705	0.999	0.966	0.998	0.950	0.986	0.839
MedViTV2-L (Manzari et al., 2026)	117.3	~26	0.953	0.910	0.999	0.987	0.943	0.716	0.999	0.973	0.999	0.961	0.987	0.851
MedLiT-nano	0.7	0.6	0.839	0.840	0.998	0.973	0.925	0.677	0.993	0.937	0.990	0.903	0.969	0.773
MedLiT-seed	2.1	1.6	0.918	0.897	0.999	0.986	0.938	0.721	0.999	0.965	0.998	0.946	0.982	0.816

Metrics marked with a dash were not reported in the study. Blue indicates the highest values for a subset while red indicates the second highest.

**Table 3:** AUC and ACC difference from MedViTV2-L.

Subset	Size	MedViTV2-L (Gheflati and Rivaz, 2022)		Difference from best model			
				MedLiT-seed		MedLiT-nano	
		AUC	ACC	AUC	ACC	AUC	ACC
RetinaMNIST	1,600	0.785	0.578	**+0.016**	**+0.020**	−0.041	−0.028
DermaMNIST	10,015	0.950	0.817	**+0.015**	**+0.013**	−0.025	−0.043
TissueMNIST	236,386	0.943	0.716	−0.005	**+0.005**	−0.018	−0.039
BloodMNIST	17,092	0.999	0.987	**0**	−0.001	−0.001	−0.014
OrganAMNIST	58,830	0.999	0.973	**0**	−0.008	−0.006	−0.036
BreastMNIST	780	0.953	0.910	−0.035	−0.013	−0.114	−0.070
OrganCMNIST	23,583	0.999	0.961	−0.001	−0.015	−0.009	−0.058
PathMNIST	107,180	0.999	0.997	−0.001	−0.024	−0.015	−0.115
ChestMNIST	112,120	0.823	0.967	−0.295	−0.033	−0.299	−0.044
OrganSMNIST	25,211	0.987	0.851	−0.005	−0.035	−0.018	−0.078
PneumoniaMNIST	5,856	0.997	0.973	−0.012	−0.082	−0.042	−0.120
OCTMNIST	109,309	0.996	0.952	−0.022	−0.093	−0.034	−0.151
ChestMNIST	112,120	0.823	0.967	−0.041	−0.138	−0.094	−0.151

From Table 2, compared to all other models, we can see that MedLiT-seed achieved highest AUC on DermaMNIST (0.965), BloodMNIST (0.999), OrganAMNIST (0.999) and RetinaMNIST (0.801). MedLiT-seed also achieved the second-highest AUC on PathMNIST (0.998) & OrganCMNIST (0.998). It remains competitive in terms of AUC, only being slightly behind MedViTV2-L for TissueMNIST and OrganSMNIST as per Table 2.

Despite their significantly smaller parameter counts—MedLiT-seed with 2.1M and MedLiT-nano with 0.75M parameters—both models demonstrated competitive or superior performance across multiple subsets. Notably, MedLiT-seed outperforms MedViTV1-L (57.6M) and ResNet-50 (23.5M) across 9 out of 12 subsets, while outputting comparable performance for BreastMNIST and PathMNIST. It even outperforms both MedViTV2-L (117M) and MedMamba-B (47.1M) on challenging subsets such as DermaMNIST and RetinaMNIST.

These results are noteworthy given that MedViT, MedMamba and other large-scale models often exceed 20M parameters, 10M in the case of MedViT and MedMamba’s tiny models, and require significantly more computational resources. MedLiT-seed’s ability to match or exceed their performance with only 2.1M parameters and a fraction of the FLOPs underscores its architectural efficiency.

MedLiT-nano, with only 0.75M parameters, consistently matched or exceeded ResNet-18 and AutoKeras across most subsets. For example, it achieved higher AUC and ACC on DermaMNIST, OCTMNIST, RetinaMNIST and BloodMNIST, and has comparable performance on PathMNIST and PneumoniaMNIST. This suggests that even the smallest variant of MedLiT is suitable for deployment in low-resource environments without sacrificing much diagnostic accuracy.

Beyond raw scale-to-performance metrics, training dynamics further highlight the advantages of MedLiT. Models pre-trained on ImageNet exhibited faster convergence and lower final training loss compared to those trained solely on MedMNIST. This was especially evident in experiments E6 and E12, where transfer learning enabled the models to start with lower initial loss and maintain stability throughout training (See Figure A6). These findings validate the use of transfer learning as a strategy to enhance generalization and reduce training time.

However, in terms of AUC, MedLiT-seed lags behind MedViTV2 and MedMamba by a few percentage points for OCTMNIST, PneumoniaMNIST, BreastMNIST and ChestMNIST. MedLiT’s accuracy for ChestMNIST appears to be the weakest of all the models, suggesting difficulty with multi-label data which will be further explored in the discussion.

## 5 Ablation study

### 5.1 Embedding size

First, we consider the effectiveness of increasing embedding size of input images on the mean testing AUC and ACC of all 12 MedMNIST 2D subsets. We evaluate this using MedLiT-nano as a baseline, where the model is solely pre-trained and fine-tuned on MedMNIST with an embedding size of 108. We compare this baseline to Experiments 9 and 10, in which the embedding size is increased to 128, and subsequently 216, under the same training configurations. Minimal differences are observed between the embedding sizes of 108 and 128, though a slightly higher Mean ACC is observed with an embedding size of 216 (see Table 4).

**Table 4:** Mean AUC and ACC for different embedding dimensions.

Experiment ID.	Embed size	Params (M)	FLOP (G)	Mean AUC	Mean ACC
E2 (MedLiT-nano)	108	0.74	0.63	0.918	0.813
E9	128	0.94	0.78	0.919	0.808
E10	216	2.11	1.59	0.924	0.823

### 5.2 Expert count

Next, we explore the effect of doubling the number of experts for the layers in each stage from 3/4/5 to 6/8/10. We fix the top-k routing parameter to 2 across all experiments to maintain constant per-token computational cost. This allows us to study whether increasing the number of experts improves performance through better parameter allocation and specialisation, rather than via increased computation. We evaluate the effects of this change under different conditions such as differing embedding sizes or training schedules (see Table A2). Interestingly, comparing each model to its own variant with an increased expert count demonstrates either a negligible increase, or even a decrease in both mean AUC and ACC (see Table 5).

**Table 5:** Mean AUC and ACC for different pre-training schedules.

Experiment ID.	Pre-training source	Epochs	Mean AUC	Mean ACC
Config-1, E10	MedMNIST pre-train	120	0.924	0.823
Config-2	ImageNet pre-train	120	0.887	0.768
Config-3, E12 (MedLiT-seed)	ImageNet pre-train	120	0.945	0.858
	ImageNet fine-tune	80		

### 5.3 Pre-training schedule

Finally, we investigate the optimal pre-training strategy for MedLiT-seed in Table 5 to achieve the best AUC and ACC across the MedMNIST subsets. We compare three pre-training schedules performed before fine-tuning on individual subsets, starting with Strategy-1 situated entirely within the medical domain with MedMNIST as the sole pre-training (E10). Config-2 introduces ImageNet as an alternative pre-training source, while Config-3 is our proposed pipeline for MedLiT-seed, involving a comprehensive pre-training and fine-tuning on ImageNet, before the model looks at MedMNIST. All other model settings such as embedding size or expert count are kept constant.

As seen in Table 6, our full pipeline achieves the highest mean AUC and ACC (Config-3, Mean AUC = 0.945, Mean ACC = 0.858), outperforming both the medical-domain baseline (Config-1, Mean AUC = 0.924, Mean ACC = 0.823) and the ImageNet pre-train configuration (Config-2, Mean AUC = 0.887, Mean ACC = 0.768). Despite the apparent domain gap between natural and medical images, ImageNet pre-training and fine-tuning appears to provide MedLiT with fundamental visual representations that transfer effectively to medical imaging tasks. MedLiT-seed learns to recognise low-level visual features and high-level structural patterns that are not exclusive to natural images. This provides a strong initialisation that medical datasets alone are unlikely to provide given their comparatively limited scale and diversity.

**Table 6:** Mean AUC and ACC for different expert counts.

Experiment ID.	Embed size	Training schedule	Expert count	Params (M)	FLOP (G)	Mean AUC	Mean ACC
E2 (MedLiT-nano)	108	MedMNIST pre-train	3/4/5	0.74	0.63	0.918	0.813
		MedMNIST fine-tune					
E8			6/8/10	0.95	0.63	0.914	0.809
E10	216	MedMNIST pre-train	3/4/5	2.11	1.59	0.924	0.823
		MedMNIST fine-tune					
E11			6/8/10	2.52	1.59	0.927	0.823
E13	216	ImageNet pre-train	3/4/5	2.11	1.59	0.944	0.861
		ImageNet fine-tune					
		MedMNIST fine-tune					
E14			6/8/10	2.52	1.59	0.943	0.845

Notably, Config-2 performs worse than Config-1 despite benefiting from ImageNet pre-training, suggesting that ImageNet pre-training alone, without a subsequent fine-tuning stage, is insufficient for effective transfer to the medical domain. We attribute this to the representations learned during ImageNet pre-training being too loosely organised for direct adaptation to medical imaging tasks via fine-tuning on the relatively small individual MedMNIST subsets. The ImageNet fine-tuning stage in Config 3 appears to serve as a critical consolidation step, structuring and refining the pre-trained features into more discriminative representations before medical fine-tuning begins. Taken together, these results justify our choice of training scheme and highlight the importance of the full staged pipeline for MedLiT-seed.

## 6 Discussion

Our results demonstrate that MedLiT can achieve excellent performance with a fraction of the parameter count and computational cost of conventional models. This finding challenges the prevailing assumption that high-performing vision transformers must be large and computationally intensive. MedLiT-seed (2.1M parameters) and MedLiT-nano (0.75M parameters) consistently deliver competitive results across a wide range of medical imaging tasks, often outperforming models that are 10–20× larger. However, the comparison against larger models should be understood in terms of the accuracy-per-FLOP trade-off rather than as a general claim of superiority.

These findings stand in contrast to the growing reliance on increasingly large foundation models, which while powerful, impose substantial computational and operational burdens. Large-scale models typically require specialized hardware, incur high inference latency, and demand extensive fine-tuning to adapt to task-specific requirements (Ding et al., 2025). Such constraints limit their practicality in real-world healthcare environments, where reliability, cost-efficiency, and responsiveness are critical. In this context, MedLiT demonstrates that carefully designed ultra-lightweight architectures can partially substitute for parameter count without sacrificing performance. MedLiT’s FLOP count is substantially lower than compared baselines, and the accuracy-per-FLOP analysis suggests it occupies a favourable position on the efficiency frontier for tasks where it performs well. We note, however, that FLOP counts are a theoretical measure, with actual inference latency depending on hardware which varies across deployment targets. We hence present the FLOP comparison as an architectural efficiency signal than a direct deployment performance guarantee.

While the present study focuses on classification benchmarks rather than deployment targets, the architectural direction MedLiT represents has longer-term implications worth considering. Large ViT models are computationally expensive at inference, requiring specialised hardware and incurring high latency and memory that might create practical friction in healthcare settings where responsiveness and cost-efficiency matter. A model family built around sparse activation and selective routing, when further optimised for inference runtimes, could in principle reduce such technical or financial barriers. Settings such as rural clinics, mobile health platforms or multi-institution deployments that cannot absorb the operational overhead of large-scale models represent a natural motivation for this line of architectural research, even if MedLiT in its current form is not yet validated for such contexts. By lowering the technical and financial barriers to deployment, ultra-lightweight models of similar architecture may also help mitigate global health disparities, especially in low- and middle-income settings where access to high-end computational resources remains limited (Chen et al., 2025).

MedLiT’s sparse MoE design is well-suited to this longer-term vision. As only a subset of experts activates per input, inference cost does not scale proportionally with total parameter count. This design supports rapid task adaptation and scalable personalization. With subsequent augmentations such as expert pruning, or fine-tuning for specific imaging modalities or diagnostic categories, this architectural foundation could support deployment of specialised model variants without incurring the cost and complexity of retraining or maintaining large monolithic models. These remain open engineering directions rather than demonstrated capabilities, but they follow naturally from the sparse routing design, representing concrete next steps toward the deployment scenarios motivating this work.

Transfer learning plays a pivotal role in MedLiT’s adaptability. Pre-training on general-purpose datasets such as ImageNet enables the model to acquire foundational visual representations, which can then be efficiently refined for medical imaging tasks. Our experiments show that this strategy improves both convergence speed and final performance, particularly when combined with domain-specific fine-tuning on MedMNIST (Figure A6). This two-stage training paradigm suggests MedLiT can adapt efficiently to new datasets, imaging modalities, or evolving clinical needs.

Despite its strengths, MedLiT exhibits limitations on multi-label tasks such as ChestMNIST, where multiple pathologies may co-occur within a single image. MedLiT achieves AUC scores comparable to ResNet but lags on accuracy. Multi-label prediction requires modelling inter-label dependencies and detecting multiple weak, spatially localized signals simultaneously. MedLiT’s reliance on independent token-level expert selection may constrain its ability to fully capture such dependencies. In addition, the current framework treats downstream tasks independently, potentially limiting opportunities for cross-task learning and shared representation. Future routing mechanisms that incorporate global image context before expert assignment may address this directly.

Limitations of the current study include the restriction to 2D MedMNIST benchmarks which, while standardised, do not capture the full complexity of clinical imaging tasks such as volumetric CT or MRI analysis. The baseline set, while representative, does not include the most recent large vision models, and direct comparison against those remains an open question. Future work should also explore multi-task training, multi-seeded uncertainty quantification and extension to higher-resolution inputs.

## 7 Conclusion

MedLiT-seed and MedLiT-nano demonstrate that sparse MoE-based routing represents a viable architectural pathway for compact medical vision models, one that achieves competitive accuracy-per-FLOP tradeoffs across diverse imaging classification tasks without relying on scale. On most MedMNIST subsets, both variants match or exceed larger baselines, supporting the premise that selective computational routing can serve as an effective design principle even on the order of 2M parameters. Where the architecture falls short, most notably on multi-label classification, the failure mode is structurally interpretable and points to concrete directions for future development. Taken together, these results suggests that the scale-performance relationship in medical vision models is more nuanced than prevailing assumptions imply, and that efficiency-oriented architectural design merits further systematic investigation.

## Data Availability

The original contributions presented in the study are included in the article/Supplementary material, further inquiries can be directed to the corresponding author. The code used to evaluate MedLiT-seed on the MedMNIST 2D datasets and reproduce the experimental results reported in this study is publicly available at: https://github.com/doppiomovimento/MedLiT.

## Footnotes

1
https://github.com/doppiomovimento/MedLiT
