# Supplementary figures and images for: Redefining lightweight vision models for healthcare AI
Source: Front Artif Intell. 2026 May 29;9:1824634. doi: 10.3389/frai.2026.1824634 (PMC13267714; doi:10.3389/frai.2026.1824634)

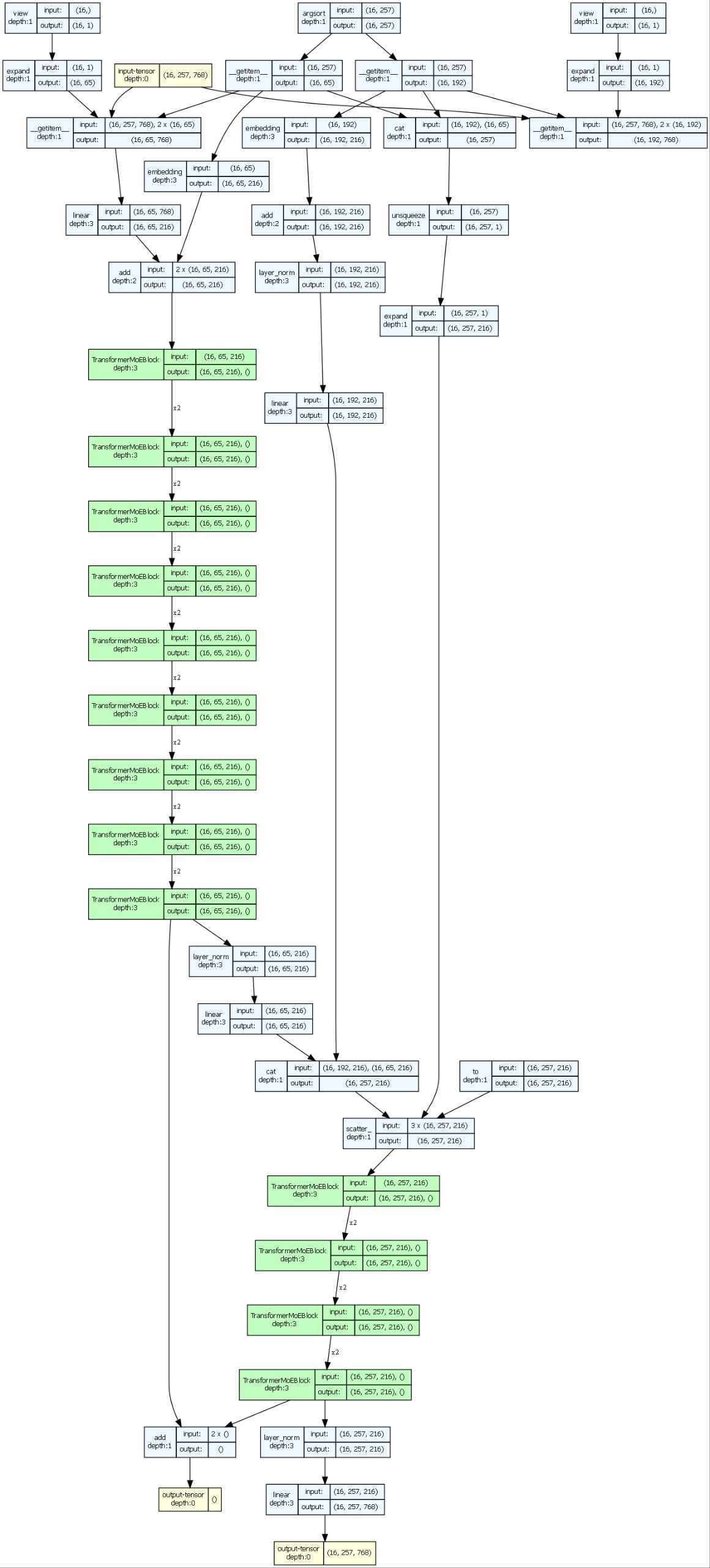

Supplement: Supplementary file 1 [file Image_1.jpg]
